# Influence of Resistance Training on Blood Pressure in Patients with Metabolic Syndrome and Menopause

**DOI:** 10.2478/hukin-2014-0093

**Published:** 2014-11-12

**Authors:** Glêbia Alexa Cardoso, Alexandre Sérgio Silva, Alesandra Araújo de Souza, Marcos Antônio Pereira dos Santos, Raquel Suelen Brito da Silva, Lavoisiana Mateus de Lacerda, Maria Paula Motae

**Affiliations:** 1 Regional University Cariri-URCA, Descentralized Unit Iguatu, Department of Physical Education.; 2 Federal University of Paraiba, Department of Physical Education, Graduate Associate Program in Physical Education University of Pernambuco/Federal University of Paraiba, Laboratory Study of Physical Training Applied to Performance and Health.; 3 Federal University of Piaui.; 4 University Mauricio de Nassau, Department of Physical Education.; 5 Departament of Sport, Trás-os-Montes de Alto Douro University, Vila Real, Portugal.

**Keywords:** cardiometabolic diseases, climacteric, post-exercise hypotension, resistance exercise

## Abstract

This study investigated the chronic and acute influence of resistance exercise on blood pressure in women with metabolic syndrome before and after climacteric. Twenty sedentary women, nine non-menopausal (RNM) and 11 menopausal (RM), performed training for 12 weeks. Meanwhile, 23 controls, 11 not menopausal (CNM) and 12 menopausal (CM), remained sedentary. Blood pressure was measured before and after the training period in conditions of rest and after a session of exercise. Training promoted variations in blood pressure at rest from 116±13 to 118±10 mmHg (p=0.73) and from 128±12 mmHg to 120±11mmHg (p=0.12) in RNM and RM, respectively. CNM and CM varied from 115±11 to 116±12 mmHg (p=0.9) and from 115±14 mmHg to 116±13 mmHg (p=0.74). Blood pressure values in one acute session did not differ between groups (p>0.05). Resistance training did not improve blood pressure in women with metabolic syndrome, regardless of climacteric.

## Introduction

Menopause is a phenomenon that is characterized by changes in lipid profile and weight gain (Agil et al., 2010). These factors, and a decrease in estrogen production, are considered risks for the development of arterial hypertension and metabolic syndrome (Agil et al., 2010; Mikkola and Clarkson, 2002; Zanesco and Antunes, 2007). In fact, lower prevalence of arterial hypertension is observed in women than men but only until menopause (Coylewright et al., 2008). More than 60% of women are affected by metabolic syndrome after climacteric (Coylewright et al., 2008; Kim et al., 2007).

It is generally known that physical exercise effectively reduces blood pressure (Kenney and Seals, 1993; Brandao-Rondon et al., 2002; Laterza et al., 2007; Laterza, Brandão-Rondon and Negrão, 2007). However, whether alterations provoked by metabolic syndrome interfere in the responses to exercise is not well-established. Likewise, whether menopause is also an intervening factor in these responses requires investigation. The bases for these assumptions include that the mechanisms whereby exercise decreases blood pressure involve a decrease in cardiac debit and peripheral vascular resistance followed by reductions in the vagal tone (Negrão and Brandão-Rondon, 2001). Investigations that demonstrate these hypotensive mechanisms are not derived from menopausal women or women with metabolic syndrome. Moreover, it is well known that at least one of these mechanisms is compromised in metabolic syndrome and menopause, which may contribute to a lower pressure response to physical treatment (Gast et al., 2011).

Acute and chronic blood pressure reductions have been demonstrated in postmenopausal women (Harvey et al., 2005; Oneda et al., 2008). However, these scarce studies were performed only with aerobic exercise prescribed. The training protocol in the only study that analyzed the blood pressure response to training with resistance exercises also included aerobic exercises, which did not allow us to affirm the impact of resistance training on lowering the blood pressure of women (Figueroa et al., 2011). Furthermore, in this study a control group of menopausal women was not included in the methodological design in order to fully determine the influence of menopause on blood pressure responses to physical exercise.

This study analyzed the influence of a resistance training protocol on resting blood pressure and blood pressure responses to a single session of exercise in non-menopausal and menopausal women with metabolic syndrome.

## Material and Methods

### Subjects

Forty-three women with metabolic syndrome participated in the study. Twenty nonmenopausal and twenty-three menopausal women were classified in accordance with the Brazilian Guidelines for Diagnosis and Treatment of Metabolic Syndrome (2005). The following inclusion criteria were applied: 1) be middle-aged; 2) have a medical certificate of clinical fitness to participate in the exercise program after orthopedic and cardiovascular, hormonal and renal examinations; and 3) perform neither physical exercise systematically nor have any sort of job that required physical effort as a professional activity before or during the study. This study excluded the women who: 1) were undergoing menopause; 2) did hormone replacement; 3) were smokers or drinkers (more than three daily doses); 4) were users of supplements or medicines, such as fibrates, statins, hypoglycemic or exogenous insulin and antiarrhythmic agents; or 5) were pregnant.

The 43 volunteers were randomly divided into four groups: resistant exercises, not menopausal (RNM - n= 9, 44±4 years), resistant exercises, menopausal (RM - n= 11, 55±8 years); control, not menopausal (CNM - n= 11, 40±4 years); and control, menopausal (CM - n= 12, 55±6 years). The sample power was previously calculated based on the study of Earth et al. (2008), who found a reduction in systolic blood pressure from 125.2 ± 9.3 mmHg to 114.7 ± 9.2 mmHg in older women who performed resistance exercises for 12 weeks, which produced an effect size of 1.22. The adoption of an alpha error of 5% and statistical power of 0.90 produced a minimum n of 8 subjects in each group in this investigation. The Ethics Committee on Human Research of the Federal University of Ceará approved this study under the protocol number 42111/2011. Each subject was informed of the risks and benefits of the study and signed an informed consent form, according to the Resolution 196/96 of the National Health Council.

### Study Design

RNM and RM groups performed a training protocol with resistance exercises that lasted 14 weeks. Blood pressure measurements were performed in exercise and control groups before and after training. Blood pressure measurements were taken in the first and last session before exercise and after a period of 60 minutes, which was determined as the recovery period after exercise for evaluation of acute blood pressure responses. Control groups participated in these two exercise sessions only.

### Adaptation to training

The subjects underwent two weeks of adaptation to the exercises that would compose the training series before the start of the study. There were two sessions each week, with a 48-hour break between sessions. The study participants performed a series of 15 repetitions for each exercise with a load that they considered easy (score between 2 and 4 on the scale of perceived exertion of OMNI - Resistance Exercise Scale (OMNI - RES)), as proposed by Gearhart et al. (2009).

### Test of 10 maximum repetitions

Twenty-four hours after the adaptation phase, a 10RM test was performed for each of the exercises in the training protocol. The test was performed based on the protocol proposed by the American College of Sports Medicine (2010). The subjects were instructed to perform 10 repetitions with a load higher than the load that was used in the adaptation phase. If a lower or higher result than proposed was obtained, a 5 min rest period was given, and the load was adjusted to a new trial. A maximum of three trials was allowed. A new test was performed 48 hours after the first test for exercises in which the load of 10RM was not reached after three attempts.

### Protocol Training

Training was conducted with exercises that worked all major muscle groups. Therefore, we adopted the following exercises: upper limbs: pulley, bench press, biceps curl and triceps French; and legs: leg extension, leg curl, squat, plantar flexion.

The training protocol was developed so that there was a progression of training loads throughout the 12 weeks. Therefore, drills were performed with one to three sets of 15 to 18 repetitions with a load of 50% of 10RM in the first four weeks so that the subjects covered 4 to 6 on the OMNI-RES scale; a 1.5 min rest period was allowed between sets and exercises. The load increased to 60% of 10RM from the 5^th^ to 8^th^ weeks, but the number of sets, repetitions, subjective perception of effort and rest periods between sets and exercises remained constant. The load progressed to 70 – 80% of 10RM from the 9^th^ through the 12^th^ weeks, the effort perception increased to 6–8 in the OMNI - RES scale, and the number of repetitions decreased to 10 to 12. The number of sets and rest periods remained constant.

### Nutritional assessment

Nutritional assessments were performed before the beginning, in the middle and at the end of the training period using a food frequency questionnaire, following the proposal of Block et al. (1986) to monitor the nutritional behavior throughout the study. AVANUTRI software, version 4.0 (Avanutri Nutrition & Computer Services, Três Rios - RJ -Brazil) for the calculation of caloric intake and macronutrients.

### Blood Pressure Measurement

Resting pressure measurements were taken 24 hours before the beginning of the adaptation to the exercise phase and 48 hours after the last session of the training protocol to evaluate the influence of the training protocol on blood pressure.

Pressure measures to a single session of exercise were performed on the first and the last session of the training protocol. Women were seated at rest for 10 minutes before exercise to take basal blood pressure. They performed the exercise session, and sat down immediately afterwards. New blood pressure measurements were taken immediately after exercise every 10 minutes during a recovery period of 60 minutes in which women remained seated.

Blood pressure was measured by auscultation using a Missouri sphygmomanometer (Embu, Brazil) purchased solely for the purposes of this research after going through a certification process for calibration. The proposal of the VI Brazilian Guidelines on Hypertension (2010) was adopted as a measurement protocol.

### Statistical analysis

The normality and homogeneity of variance in the data were analyzed using the Shapiro- Wilks and Levene tests, respectively. A one-way ANOVA was used to compare the pre-intervention values between groups. A two-way ANOVA was used to analyze changes in cardiovascular variables with groups and time points (before and after intervention) as factors. For all analyses, p<0.05 was adopted as statistically significant, and post-hoc comparisons were performed using the Newman-Keuls test, when necessary. Data are presented as means ± standard deviation.

## Results

All subjects had hypertension as a component of their metabolic syndromes. They were previous users of antihypertensive medications, such as angiotensin converting enzyme (IACE), beta-blockers and diuretics, and they remained on the same medications and dosages throughout the study. The women showed controlled blood pressure before starting the study despite being hypertensive. The menopausal women in the control and experimental groups showed levels of total lipoproteins and fasting glucose above the upper limit recommended by the III Brazilian Guidelines on Dyslipidemia (2001) and Guidelines for the Treatment and Monitoring of Diabetes Mellitus (2008), but the values did not differ significantly compared to the non-menopausal groups (Table 1). No significant difference was observed with regard to body mass, waist circumference, and blood pressure between the four groups. CNM, CM, RNM and RM presented similar nutritional intake at the beginning of the intervention with resistance exercises, and no group changed their consumption of total calories and macronutrients after the training period (Table 1).

**Figure 1:**
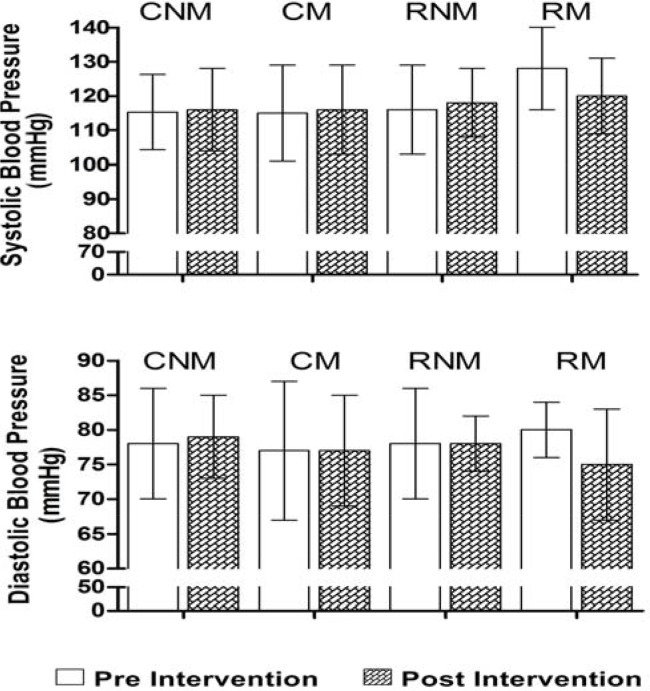
Resting systolic and diastolic pressure during pre-intervention (white bars) and post-intervention of 12 weeks of training with resistance exercises (drawn bars) in menopausal and non-menopausal subjects. Data are presented as means and standard deviations. There were no significant differences in any of the variables when treated by one-way ANOVA.

Figure 1 presents resting blood pressures before and after intervention with resistance training. Only the RM group showed a slight decrease in SBP (from 128 ± 12 mm Hg to 120 ± 11mmHg, p > 0.05) and DBP (from 80 ± 4 mmHg to 75 ± 8 mmHg, p > 0.05) in resting conditions. There was no significant difference between these values before and after training at the end of the training intervention, and the values of resting blood pressure of this group did not differ from the other groups.

**Figure 2:**
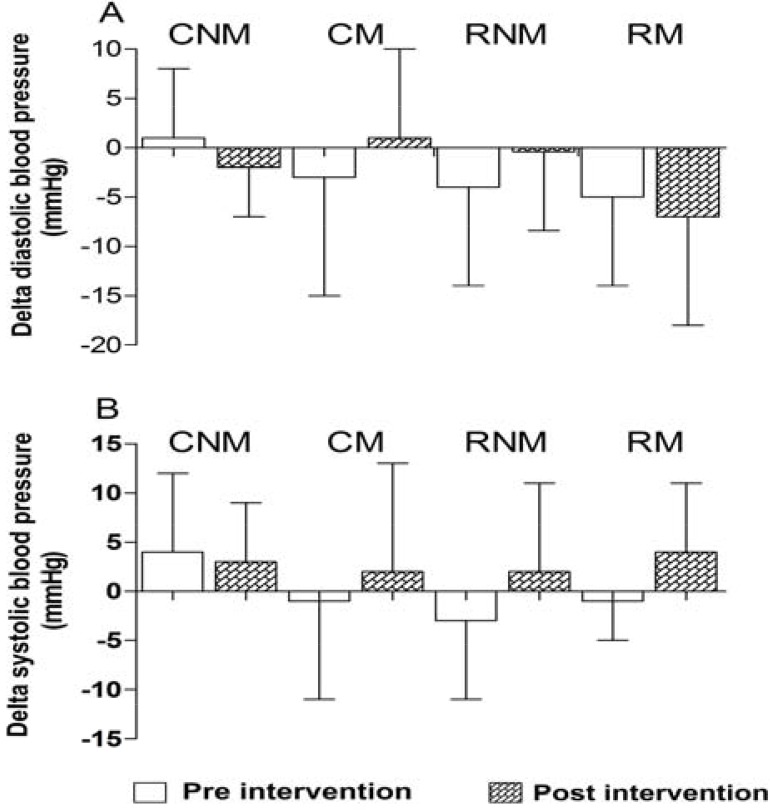
Delta systolic blood pressure (panel A) and diastolic (panel B) between the moments of rest and after a single session of resistance exercises performed before (white bars) and after (hatched bars) 12 weeks of a training program with resistance training in menopausal and non-menopausal women. Data are presented as means and standard deviations. There were no significant differences in any of the variables using one-way ANOVA.

Figure 2 shows the pressure responses to two isolated resistance exercise sessions, before the start and at the end of the intervention protocol with resistance training. CM, RNM and RM demonstrated systolic hypotensive behaviors in the pre-intervention session of −3 ± 12 mmHg, −4 and −5 ± 10 mmHg ± 9 mmHg, respectively, but no significant difference between pre- and post-exercise was noted. CNM showed no systolic hypotension between the basal and post-exercise time points. CNM and RNM demonstrated systolic hypotensive behavior after exercise intervention of −2 ± 5mmHg and −7 ± 11mmHg, p > 0.05, respectively, but no significant difference was observed.

CM, RNM and RM showed a slight post-exercise hypotension in DBP at the pre-training intervention (−1 ± 10 mmHg, −3 ± 8 mmHg and − 1 ± 4 mmHg, respectively) as shown in Figure 2 (panel B). Only the CNM group showed no reduction in pressure in this exercise session, which is similar to the systolic component. No reduction in DBP was observed at the end of the training protocol in the control or exercise groups.

## Discussion

The results of this study indicate that a training program with resistance exercises does not reduce blood pressure significantly in women with metabolic syndrome regardless of menopause status. A single session of resistance exercise modestly promoted an acute reduction in blood pressure in these women, and the implementation of a training program did not affect this response.

Cornelissen and Fagard (2005) published a meta-analysis of nine randomized studies that indicated a reduction of 3.2 mmHg and 3.5 mmHg in systolic and diastolic blood pressure after intervention with resistance training in men and women with hypertension who were not affected by metabolic syndrome. These values are low compared to the blood pressure reduction of 11 mmHg in systolic blood pressure and 8 mmHg in diastolic blood pressure that were induced by aerobic exercise training (Hagberg et al., 2000). However, pressure decreases after resistance training protocol are not always observed after 12 weeks of training, Moraes et al. (2011) found no significant reduction in blood pressure.

Figueroa et al. (2011) verified a chronic attenuation in systolic and diastolic blood pressures in menopausal women, but the training protocol included both resistance and aerobic exercise. No studies of training protocols that used only resistance exercises have been published. Only Egaña et al. (2010) conducted research in post-menopausal women with metabolic syndrome who underwent 12 weeks of training with resistance exercises using elastic bands to replace the traditional machines for this type of exercise. They found an increase in the blood flow, but they did not report whether this phenomenon was followed by a reduction in blood pressure. Therefore, whether the state of menopause and metabolic syndrome interferes in blood pressure responses to training with resistance exercises is not yet properly understood.

We did not find significant chronic alterations in blood pressure in response to the adopted training protocol, but we observed a blood pressure decrease from 7 mmHg to 9 mmHg and from 1 mmHg to 9 mmHg in systolic and diastolic blood pressure, respectively, in the group of menopausal women who participated in the physical training program. This reduction was not significant, but the average values were higher than the meta-analysis of Cornelissen and Fagard (2005) (−3.2 mmHg and −3.5 mmHg for systolic and diastolic values, respectively). Studies of aerobic exercise, in which acute blood pressure reductions are much more evident, noted that standard deviations tended to be high (Grizzo et al., 2011; Rodriguez et al., 2011). However, these authors perceive the blood pressure reductions as clinically significant. Therefore, one cannot completely disregard the clinical relevance of small pressure reductions (not statistically significant) that were promoted by exercise. Chobanian et al. (2003) reported that reductions as small as 2 mmHg in blood pressure were associated with a 6% reduction in the incidence of mortality from the stroke and 4% for coronary artery disease.

Our sample was composed of subjects with hypertension, but the blood pressure values in the pre-intervention were close to normal values. Laterza et al. (2007) indicated that the magnitude of blood pressure reductions in response to exercise training was higher in subjects with higher initial values, and hypertensive subjects, who had values close to normal, tended to have lower pressure reductions in response to exercise. Indeed, studies that showed hypotensive effects of resistance exercise included women who started with initial systolic blood pressure values of 125±1.8 mmHg to 150±13 mmHg and diastolic values of 76±1.4 mmHg to 93±5 mmHg (Cornelissen and Fagard, 2005; Moraes et al., 2011), which were much higher than the values in this study. We emphasize that the RM group in our study and the best prospect to have chronic systolic blood pressure reduction, and this group had the highest blood pressure value before the study began.

The training protocol was updated to include a progression of charges in loads over the 12 weeks of training. The adoption of the effort subjective perception scale of OMNI-RES assured that the intensity was updated according to the strength gains obtained. Therefore, we believe that the absence of a significant reduction in resting blood pressure did not happen due to failures in the adequacy of the training load over 12 weeks.

It is well-established that reductions in blood pressure occur during the first minutes after a single exercise session compared to basal levels (Kenney and Seals, 1993; Laterza et al., 2007; Mota et al., 2011). These decreases are seen as sub-acute, and they are named post-exercise hypotension (PEH) (Kenney and Seals, 1993). Two previous studies showed a reduction of 5 mmHg and 22 mmHg for SBP and 2 and 16 mmHg for DBP in hypertensive men and women, respectively, but not in subjects with metabolic syndrome or menopause (Mota et al., 2009; Veloso et al., 2010). The reduction in blood pressure in our study was not significant, but we observed a hypotensive magnitude in the CM, RNM and RM groups that was in line with the results of two previous investigations in which the authors stated that the exercise that afforded a hypotensive effect was the session held before the start of the training protocol.

The single session performed after 12 weeks of training resulted in sub-acute pressure responses similar to the pre-training session, which indicates that the exercise program did not affect post-exercise hypotension. The inclusion of sub-acute blood pressure responses to an exercise session in this study was motivated by the fact that, although post-exercise hypotension is a well-established phenomenon, the effects of training on this response are not known. A reduction in blood pressure at rest is usually observed after a training protocol, but we expected that sub-acute blood pressure reduction levels were diminished due to the lower basal values of blood pressure. However, the absence of post-exercise hypotension prevented us from answering whether chronic blood pressure reductions in resting blood really minimized the magnitude of post-exercise hypotension.

These considerations suggest that the initial level of blood pressure is a variable that should be taken into account in future studies of physical training. Therefore, we recommend that future studies assess the effectiveness of resistance exercise training by forming subgroups of hypertensive subjects who have initial blood pressure values above and below 140/90 mmHg, which are the borderline values for determining whether blood pressure is controlled.

## Conclusions

Previous studies verified chronic reductions in the blood pressure, but our study using resistance exercise training did not promote chronic reductions in blood pressure in women with metabolic syndrome. The condition of menopause was not an influential variable on blood pressure responses to exercise. Despite the absence of chronic blood pressure reductions, data from this study indicate a modest capacity of resistance exercise to promote acute reductions in blood pressure in women with metabolic syndrome.

## Figures and Tables

**Table 1:** Anthropometric, biochemical characteristics, basal hemodynamic and nutritional behavior before and after training of non-menopausal and menopausal subjects.

	CNM	CM	RNM	RM
n	11	12	9	11
Age (years)	44±4	55±8	40±4	55±6
Body Mass (kg)	73±11	74±14	70±11	67±15
Body Mass Index (kg/m^2^)	31±4	31±4	30±5	29±6
Waist circumference (cm)	101±8	102±9	95±9	97±12
Total lipoprotein (mg/dl)	190±29	209±56	193±43	213±34
High Density	38± 15	38±16	44±17	36±15
Lipoproteins (mg/dl)				
Triglycerides (mg/dl)	138±13	180±20	153±80	169±115
Glucose (mg/dl)	90±10	103±35	85±8	115±55
Systolic Blood Pressure (mmHg)	115±11	115±14	116±13	128±12
Diastolic Blood Pressure (mmHg)	78±8	77±10	78±8	80±4
Caloric intake	1703±555	1555±319	1802±340	1720±455
Pre-training Carbohydrates	53,8±14	54±14	55,0±13	54,7±17
Lipids	27,1±8	27±9	29,3±12	28,7±9
Proteins	19,1±6	19±6	15,7±9	16,6±9
Caloric intake after training Carbohydrates	1810±508	1740±435	1845±400	1720±420
	54,0±14	55±15	55,3±12	55,2±15
Lipids	28,5±5	27±8	26,9±7	27,9±8
Proteins	18±5	18±7	17,8±6	16,9±7

Data are presented as means and standard deviations of the mean. There were no significant differences between any of the variables. The data were treated by means of one-way ANOVA, except the variables of nutritional intake where two-way ANOVA was applied.
